# Explaining inconsistencies between data on condom use and condom sales

**DOI:** 10.1186/1472-6963-5-5

**Published:** 2005-01-15

**Authors:** Dominique Meekers, Ronan Van Rossem

**Affiliations:** 1 Department of International Health and Development, School of Public Health and Tropical Medicine, Tulane University, 1440 Canal Street, Suite 2200, New Orleans, LA 70112, USA; 2 Department of Sociology, Ghent University, Universiteitstraat 4, 9000 Gent, Belgium

## Abstract

**Background:**

Several HIV prevention programs use data on condom sales and survey-based data on condom prevalence to monitor progress. However, such indicators are not always consistent. This paper aims to explain these inconsistencies and to assess whether the number of sex acts and the number of condoms used can be estimated from survey data. This would be useful for program managers, as it would enable estimation of the number of condoms needed for different target groups.

**Methods:**

We use data from six Demographic and Health Surveys to estimate the total annual number of sex acts and number of condoms used. Estimates of the number of sex acts are based on self-reported coital frequency, the proportion reporting intercourse the previous day, and survival methods. Estimates of the number of condoms used are based on self-reported frequency of use, the proportion reporting condom use the previous day and in last intercourse. The estimated number of condoms used is then compared with reported data on condom sales and distribution.

**Results:**

Analysis of data on the annual number of condoms sold and distributed to the trade reveals very erratic patterns, which reflect stock-ups at various levels in the distribution chain. Consequently, condom sales data are a very poor indicator of the level of condom use. Estimates of both the number of sexual acts and the number of condoms used vary enormously based on the estimation method used. For several surveys, the highest estimate of the annual number of condoms used is tenfold that of the lowest estimate.

**Conclusions:**

Condom sales to the trade are a poor indicator of levels of condom use, and are therefore insufficient to monitor HIV prevention programs. While survey data on condom prevalence allow more detailed monitoring, converting such data to an estimated number of sex acts and condoms used is not straightforward. The estimation methods yield widely different results, and it is impossible to determine which method is most accurate. Until the reliability of these various estimation methods can be established, estimating the annual number of condoms used from survey data will not be feasible. Collecting survey data on the number of sex acts and the number of condoms used in a fixed time period may enable the calculation of more reliable estimates of the number of sex acts and condoms used.

## Background

Programs that promote condom use for HIV prevention typically monitor their progress through survey-based indicators, such as the percentage of the population who ever used a condom or the percentage who used a condom in their last sex act with a casual or regular partner [1,2]. Such information is routinely collected in national surveys, such as the Demographic and Health Surveys (DHS) and the CDC Reproductive Health Surveys [3,4]. In addition, HIV prevention programs often monitor the number of condoms sold and/or the number distributed free of charge.

The purpose of this study is to explain inconsistencies between information on reported levels of condom use and data on the number of condoms sold and distributed. Understanding the apparent inconsistencies between sales and survey data will help clarify to what extent the concerns about condom wastage, misreporting, and other related problems are founded. It will also provide guidance for improving the monitoring of condom sales and distribution, and for improving survey questionnaires. To achieve these objectives, we use survey data from six Demographic and Health Surveys to estimate the total annual number of sex acts in a country, and the total number of condoms used in those sex acts, and compare the totals with reported data on condom sales and distribution.

At least in some instances, survey information on condom use and condom sales records appear to be inconsistent [5,6]. For example, in some countries we observe steady increases in reported condom sales while survey indicators suggest that there has been no significant increase in the percentage of condom use in last sex across survey rounds. In Zimbabwe, sales of socially marketed *Protector Plus *condoms increased from 1.9 million in 1997, to 4.8 million in 1998, to 8.9 million in 1999. Data on public sector condom distribution, which we discuss later in this paper, suggest that public sector sales also increased substantially. Yet, nationally representative surveys indicate that condom use in last sex stayed constant between 1996 and 1999 at roughly 34% for males and 17% for females [6,7]. Similarly, in Tanzania, sales of socially marketed *Salama *condoms increased steadily between 1995 and 2000, as did condom distribution by the Ministry of Health. However, survey data indicate that condom use at last intercourse remained roughly constant between 1996 and 1999, for both men and women [5]. These discrepancies suggest that either the data on reported levels of condom use or the data on condom sales and distribution are inaccurate, or possibly that both are inaccurate.

Inaccuracies in the number of condoms sold or distributed are likely because sales figures typically represent sales to the trade (i.e., sales to wholesalers and distributors) rather than sales to consumers. Consequently, the recorded sales numbers will include condoms that are being stocked at various levels of the distribution chain. In addition, some of the condoms that are sold and/or distributed may be wasted or smuggled to other countries.

In addition to these potential problems with condom sales data, there are concerns that reported condom use in surveys may be inaccurate. For example, there are concerns that respondents may overreport condom use because they do not want to admit to the interviewer that they are engaging in risky sexual behavior. There are also concerns that condom use may be underreported because condoms are frequently used with sex workers, which stigmatizes condom use. Women may also underreport condom use because it is a male method. Some questionnaires try to overcome this by asking "The last time you had intercourse, was a condom used?" rather than "The last time you had intercourse, did you use a condom?" [3].

## Methods

### Sources of data

This study uses two types of data: data on condom sales and distribution, and survey data on self-reported condom use. We restrict our analysis to data from four countries in sub-Saharan Africa (Kenya, Tanzania, Nigeria, and Zimbabwe), largely because these countries have strong condom social marketing programs and therefore relatively good data on condom sales and distribution. In addition, Tanzania and Zimbabwe are two of the countries where discrepancies between condom distribution and condom use have been noted.

Data on sales of socially marketed condoms were obtained from DKT International's Social Marketing Statistics [8-14], while data on donor-supplied public sector condoms were obtained from UNFPA and USAID [15,16]. Data on commercial condom sales are not readily available, but for recent years very rough estimates were obtained from Population Services International's MIS database [17]. As commercial sales tend to be negligible in the countries under consideration, the lack of accurate data on commercial sales is unlikely to have a significant effect on our findings.

The survey data used in this study include the following Demographic and Health Surveys (DHS): Kenya (1998), Nigeria (1999), Tanzania (1996, 1999), and Zimbabwe (1994, 1999). Each of the six surveys comprises a representative sample of females aged 15–49 and of males 15–54 (note that the upper age limit varies for men, see Table 1). For more detailed information on the sampling methods and the data collection, we refer the reader to the DHS reports for these surveys [18-23].

**Table 1:** Data available in selected DHS surveys on frequency of intercourse and probability of condom use

Country	Year	Sex	Age range	Time since last intercourse	Frequency of intercourse	Condom use during last intercourse	Frequency of condom use
Kenya	1998	Men	15–54				
		Women	15–49				

Nigeria	1999	Men	15–64				
		Women	15–49^1^				

Tanzania	1996	Men	15–54				
		Women	15–49				
	1999	Men	15–59				
		Women	15–49				

Zimbabwe	1994	Men	15–54				
		Women	15–49				
	1999	Men	15–54				
		Women	15–49				

Note: ^1 ^The age range for women in the 1999 NDHS is 10 to 49. To enhance comparability, we restricted our analysis to women aged 15 to 49.

Determining the total annual number of condoms used in a population requires information on the frequency of intercourse. Unfortunately, recent sexual behavior surveys typically do not allow the quantification of the number of sex acts [24].

While some of the DHS surveys from the late 1980s and early 1990s did ask respondents about the frequency of intercourse in a fixed time interval (e.g., frequency of intercourse in the past month), such a question has not been included in recent surveys [25]. For example, the standard questionnaire for DHS surveys implemented since 1997 does not include a question on the frequency of intercourse. In the surveys included in our study, the 1994 Zimbabwe survey was the only one that included a question on the self-reported frequency of intercourse (see Table 1). However, the DHS surveys do ask respondents about the time since they last had intercourse [3,26]. Hence, our analysis estimates the total annual number of sex acts on the basis of reported data on time since last intercourse [5,27,28]. Depending on the survey, it may or may not be possible to differentiate the frequency of intercourse by partner type. Differentiation by partner type may be important, as it is believed that men who admit having a nonmarital partner are unlikely to underreport the frequency of intercourse [24].

All DHS surveys asked whether respondents used a condom in their last sex act. We use this information to estimate the probability of condom use, and, subsequently, to estimate the total annual number of condoms used in the country.

### General estimation procedure

In theory, estimating the total number of condoms used in a population is straightforward. The estimated mean number of condoms used per sexually active person (*C*) equals the product of the frequency of intercourse, or the number of sex acts (*F*), and of the probability of condom use (*p*):

*C *= *F *× *p *    (1)

The total number of condoms used (*C*^*T*^) then can be calculated by multiplying *C *with the proportion of individuals who are sexually active (*s*) in the population at risk and with size of the population at risk (*N*):

*C*^*T *^= *N *× *s *× *C *    (2)

Since the frequency of intercourse and the probability of condom use are known to vary by age and marital status [25,27,29-32], it is advisable to estimate these coefficients separately for various subpopulations and subsequently to calculate a weighted average for the entire population. In this paper, we stratified our estimates by the respondents' age and marital status. The formula to calculate the mean annual number of condoms used per sexually active respondent is:



where *w*_*a *_is the weight for age group *a*, *m*_*a *_and (1 - *m*_*a*_) are the proportion of married and unmarried respondents in age category *a*, and *s*_*am *_and *s*_*au *_are the proportion of sexually actives where the subscripts *am *and *au *refer to the rates for married and unmarried respondents in age category *a*, respectively.

We used five-year age categories, and based the age weights on the age distribution within the household file of the DHS, as no other reliable data on the age structure of the population in these countries were available (preliminary analyses with one-year age groups produced similar results). Marital status and the marital status weights were derived from the individual respondent files of the DHS. Following the DHS definition, we define marriage as formal marriage or living together. Information on current sexual activity, defined as having had sex at least once in the past year, was also obtained from the individual respondent files of the DHS. Data on the countries' population size were obtained from the 2003 World Bank World Development Indicators and are summarized in Appendix [see Additional File 1].

Although the above procedure is simple, data on the two main components, *F *and *p *are not readily available and need to be estimated. The following sections describe the procedures for estimating them.

### Methods for estimating frequency of intercourse

This section describes methods to estimate frequency of intercourse. Three types of estimation methods are presented: 1) estimation based on the reported frequency of intercourse during a four-week period, 2) methods based on the proportion of respondents reporting intercourse the day before the interview, and 3) survival analyses based on the time since last intercourse.

All methods follow a similar strategy: 1) Estimate the mean likelihood or frequency of intercourse for a specific time unit (e.g., for a day, one week, or four weeks) for each of the subpopulations, and 2) estimate the mean frequency of intercourse per year for the entire population by calculating a weighted average of the subpopulation results. The general formula is:



where *F*_*i *_stands for the annual frequency of intercourse estimated by method *i*, *f*_*iam *_and *f*_*iau *_for the estimated mean likelihood or frequency of intercourse per time unit using method *i *for married and unmarried persons in age category *a*, respectively, and *n*_*i *_the number of time units for this method in a year.

Some surveys asked married respondents separate questions about the time since last intercourse with the respondents' spouse and with the respondents' other partners. Such questions were included in the 1998 Kenya and 1996 Tanzania DHS surveys. For these surveys, the formula becomes:



where the *b *subscript in *F*_*ib *_indicates that for married respondents marital and extramarital sex were included separately.

#### Method F_1_

When self-reported data on the frequency of intercourse during the past four weeks are available, such as in the 1994 Zimbabwe DHS survey, the annual number of sex acts can be estimated by extrapolation. Assuming the past four weeks are representative of the respondents' behavior, the mean annual number of sex acts can be estimated by multiplying this four-week frequency with 13 (*n*_1 _= 13). However, because few recent surveys contain this type of information, it is generally necessary to use other estimation methods.

#### Method F_2_

The frequency of intercourse can be estimated on the basis of the proportion of respondents reporting intercourse the day before the interview [5]. Assume each of a group of individuals has 104 sex acts per calendar year (i.e., two sex acts per week). Assuming one sex act per day that intercourse occurs, the probability of intercourse on any given day during the calendar year would equal 104/365, or 0.285. Hence, it is expected that, on average, 28.5% of the population will have intercourse on any given day.

In other words, the proportion of the population reporting intercourse on any given day equals the daily probability of intercourse. Therefore, the annual number of sex acts can be estimated by multiplying the proportion of respondents who had intercourse the day before the interview by 365.

The advantage of this method is that it is simple to calculate, and that use of data that refer to the day before the interview minimizes recall problems. The disadvantage is that the method does not take into account that some people may have more than one sex act in a day (i.e., only one of those sex acts will be counted), so that the frequency of intercourse may be slightly underestimated. In turn, the impact of this more frequent intercourse on condom use may be somewhat greater than results would indicate, as the uncounted numbers may represent commercial sex workers with a relatively high condom use.

Another problem with this method is that for some surveys the percentage of respondents reporting last having intercourse the day before the survey does not appear to be reliable. For example, in the 1998 Kenya survey the percentage of respondents reporting last having sex one day before the survey was smaller than the percentage last having sex two days before the survey (4.1% vs. 8.9%). Similarly, in the 1999 Nigeria survey 0.7% reported last having intercourse one day before the survey, compared to 10.0% who reported having sex two days before the survey. In the other surveys, the percentage reporting last having sex the day before the survey is slightly higher than the percentage last having sex two days before the survey. While it is unclear why so few respondents in the Kenya and Nigeria surveys reported last having intercourse the day before the survey, the implication is that the *F*_2 _estimates for these surveys appear to be unrealistically low.

#### Method F_3_

A third alternative is to estimate frequency of intercourse based on data on the duration since last intercourse, which is collected in all DHS surveys [27,28]. This group of techniques is based on the fact that mean duration between two successive acts of intercourse provides an estimate of the frequency of intercourse. The major difficulty with this approach is that the duration between two successive sex acts is a closed interval, while the available data – duration since last intercourse – is an open interval.

Slaymaker and Zaba [28] deal with this inconsistency by using survival analyses with an exponential decay function. The survival analysis estimates the daily probability of intercourse. The estimated annual number of sex acts is obtained by multiplying the average daily probability of intercourse by 365.

One of the main weaknesses of this approach is the assumption that daily probability of intercourse is constant and can be estimated with an exponential decay function. Since data on the actual distribution of the intervals between two successive sex acts are not available in DHS surveys, one cannot determine whether the exponential decay function provides a good fit for the data. Using a function that does not match the data well would introduce a very large error in the estimated annual number sex acts (and consequently in the estimated number of condoms used), rendering the results meaningless.

### Methods for estimating the probability of condom use

As most DHS surveys only contain data on whether a condom was used in the respondent's last intercourse, we must assume that condom use at last sex is typical for the likelihood of condom use for a given subpopulation. Three different estimations for the likelihood of condom use are explored in this paper, two of which are based on data on condom use at last intercourse and one of which is based on the self-reported frequency of condom use.

#### Method p_1_

For surveys that collected information on the frequency of condom use, this information can also be used to estimate the probability of condom use. Unfortunately, none of the DHS surveys asked direct questions about both the number of sex acts and the number of condoms used (for an example of a survey that collects such data, see [33]. However, some DHS surveys did ask respondents how frequently they used condoms. For example, the 1994 Zimbabwe DHS first established how often respondents had sex with their spouse and other partners in the past four weeks. Next, respondents were asked, "Was a condom used on any of these occasions?" Respondents who answered that a condom was used were asked, "Was it each time or sometimes?" Hence the frequency of condom use was coded as "Yes, each time," "Yes, sometimes," or "Never." To obtain an estimate for the probability of condom use for each of these categories, we cross-tabulated this reported frequency of condom use against condom use in last intercourse. The results showed that 93% of men claiming to always use condoms reported using a condom in last intercourse. Similarly, 44% of those claiming to sometimes use condoms and 2% of those claiming to never use condoms reported that they had used a condom in last intercourse. Thus, we recoded the three categories for frequency of condom use among men as 0.93, 0.44, and 0.02. For women, the values were 0.94, 0.47, and 0.01, respectively. The probability of condom use was then calculated as the mean value for each of the sub-samples.

#### Method p_2_

The first estimate of the probability of condom use simply equals the proportion of a sub-sample (by age and marital status) who reported using a condom at last intercourse. This estimate was also used by Collumbien et al. [24]. Information on condom use in last intercourse is available in all DHS surveys. For surveys that collected data on condom use at last intercourse by partner type, such as the 1998 Kenya and 1996 Tanzania DHS surveys, taking this information into account can refine the estimate of the probability of condom use.

#### Method p_3_

An alternative measure of the probability of condom use equals the proportion of respondents who reported using a condom at last sex among those who had sex the previous day. This indicator has the advantage that it is less likely to be subject to recall errors. It also avoids the problem that condom use at last intercourse may be dependent on the time since last intercourse. However, this measure has the disadvantage that it tends to be less reliable because it is based on information from a much smaller number of observations (those reporting intercourse the day before the interview).

### Estimating the annual number of condoms used

We estimate the annual number of condoms used by multiplying the annual number of sex acts with the probability of condom use for each of the strata by age and marital status, as described in Equation 3. Because we have three different methods to estimate the annual number of sex acts and three methods to estimate the probability of condom use, up to nine estimates of the annual number of condoms used are provided, depending on the available data. Moreover, separate estimates were calculated using data from the female and male DHS surveys, as there are known gender differences in the reported frequency of intercourse and levels of condom use [30,32,34].

## Results

### Reported condom sales and distribution

**Figure 1:**
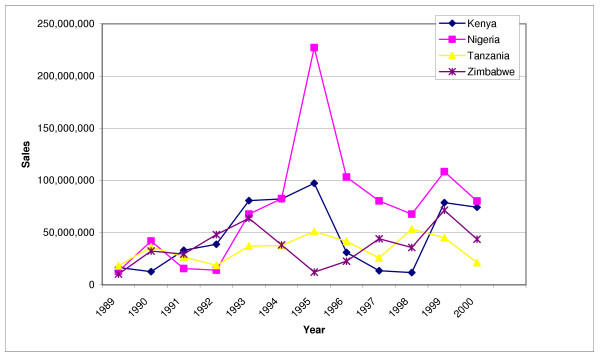
Annual number of condoms sold and distributed, by country

Figure 1 shows trends in the annual number of condoms sold or distributed in Kenya, Nigeria, Tanzania, and Zimbabwe. Although these statistics represent the number of condoms sold or distributed to the trade (i.e., to distributors, wholesalers, and retailers), it is often assumed that they will mimic sales to consumers, because the trade is unlikely to re-stock unless there is sufficient consumer demand.

Figure 1 reveals very erratic patterns in the number of condoms sold or distributed in each of the four countries. The most dramatic pattern is observed for Nigeria. The total number of condoms distributed in Nigeria increased from 13 million in 1989 to 42 million in 1990, but then declined to 14 million in 1992. Between 1992 and 1994, condom distribution increased rapidly to 83 million, and by 1995, Nigerian condom sales jumped to 227 million. However, the very next year the number of condoms distributed dropped back to 103 million and continued to decline to 68 million in 1998. In 1999, condom sales rapidly increased to 108 million. The trend in the number of condoms distributed in Kenya is equally erratic. In Kenya, the total number of condoms distributed increased rapidly from 17 million in 1989 to 39 million in 1992, to 97 million in 1995. However, from 1996 onward, the number of condoms distributed dropped dramatically, to reach only 12 million in 1998. By 1999, condom distribution jumped to 79 million. The number of condoms distributed in Tanzania and Zimbabwe is considerably lower, but also shows very large year-to-year fluctuations.

It is clear that these drastic fluctuations in the number of condoms sold or distributed do not reflect real differences in the level of condom use, as this would require major changes in behavior (and behavior is known to change very slowly). Since statistics on the number of condoms sold or distributed reflect sales to the trade, not consumers, it is highly likely that the observed fluctuations in the number of condoms distributed simply reflect fluctuations in condom inventory due to a stock-up of condoms at one or more levels of the distribution system, the addition of new condom outlets, and so on. For example, data from condom distribution surveys in Kenya indicate that the percentage of retail outlets that were selling socially marketed *Trust *condoms increased from 25% in 1998 to 32% in 1999. Similarly, the percentage of retail outlets selling public sector condoms increased from 2% to 6%. The percentage of retail outlets selling other brands stayed constant at 3% [35,36]. Assuming that outlets sell only one type of condoms, the percentage of retail outlets selling any type of condom increased from 30% to 41%, which implies that that the total number of retail outlets that sell condoms may have increased by as much as 37% (= 41/30 * 100) in just one year. Such an increase in the number of retail outlets that carry condoms would require a substantial increase in the number of condoms sold to the trade in order to fill the pipeline (i.e., to supply national and regional distributors, wholesalers, and retailers).

In addition, our estimates of the number of public sector condoms are not the actual number of public sector condoms distributed to the population, but rather the total number of condoms provided to each country by international donors. It is possible that many of these condoms are still stocked at Ministry of Health warehouses and similar distribution hubs, or at local health clinics. The actual number of public sector condoms that reach the hands of consumers is unknown. Therefore, the data that are available on the number of condoms that have been sold or distributed seem to provide an estimate of the total of number of condoms that were in circulation during the course of the year, rather than the number provided to consumers.

In other words, the current data on the number of condoms sold or distributed provide a very poor estimate of the actual number of condoms used. For example, as shown in Figure 1, condom distribution in Nigeria peaked at 227 million in 1995. However, condom distribution subsequently dropped to a level far below that of the period preceding the peak. This drop-off in sales to the trade between 1995 and 1997 suggests that some of the 227 million condoms sold to the trade in 1995 were not sold to consumers until 1996 or 1997, if not later. Hence, changes in condom sales do not necessarily indicate any changes in condom use. Measuring changes in the level of condom use requires either collecting data on retail sales, which is not feasible in most developing countries, or using sample surveys to measure the level of condom use.

### Estimated annual number of sex acts

Table 2 summarizes the results of different estimates for the mean annual frequency of intercourse for both male and female samples in the six DHS surveys used. We first discuss the results from the 1994 Zimbabwe DHS survey, for which all three methods for estimating the per capita annual number of sex acts could be calculated. Hence, these data are ideal for comparing the estimate based on self-reported data, *F*_1_, with the two estimates based on the duration since last intercourse (*F*_2 _and *F*_3_). Next, we discuss the results for the other surveys, for which only methods *F*_2 _and *F*_3 _could be estimated.

**Table 2:** Estimated annual number of sex acts (mean number per sexually experienced respondent)

Country	Year	Sex	Marital Status	N of Cases	Proportion Currently Sexually Active	Estimation Method		
						
						Self-Reported Coital Frequency (F_1)_	Proportion Having Sex Previous Day (F_2)_	Survival Analysis, Constant Hazard (F_3)_
Kenya	1998	Men	Unmarried	1,644	66.1%	-.-	4.8	6.2
			Married	1,763	98.2%	-.-	22.4	16.3
			All	3,407	82.7%	-.-	15.8	12.4
		
		Women	Unmarried	3,034	40.3%	-.-	0.9	2.6
			Married	4,847	93.5%	-.-	16.5	9.2
			All	7,881	73.0%	-.-	13.2	7.8

Nigeria	1999	Men	Unmarried	1,072	42.9%	-.-	0.8	5.6
			Married	1,608	92.0%	-.-	3.4	7.2
			All	2,680	72.4%	-.-	2.7	6.8
		
		Women	Unmarried	4,002	34.5%	-.-	2.2	3.5
			Married	5,808	82.0%	-.-	6.2	4.6
			All	9,810	67.8%	-.-	5.6	4.5

Tanzania	1996	Men	Unmarried	985	43.0%	-.-	13.8	8.7
			Married	1,268	92.0%	-.-	51.8	7.9
			All	2,256	70.6%	-.-	41.6	8.1
		
		Women	Unmarried	2,715	31.2%	-.-	14.2	5.3
			Married	5,404	86.2%	-.-	49.7	5.5
			All	8,120	67.8%	-.-	44.2	5.4
		
	1999	Men	Unmarried	1,544	57.6%	-.-	7.0	5.0
			Married	1,998	98.1%	-.-	48.9	15.6
			All	3,542	80.5%	-.-	35.9	12.3
		
		Women	Unmarried	1,421	47.0%	-.-	7.7	3.6
			Married	2,608	96.7%	-.-	48.5	10.2
			All	4,029	79.2%	-.-	39.9	8.9

Zimbabwe	1994	Men	Unmarried	1,126	53.3%	20.9	8.4	4.2
			Married	1,015	99.3%	81.9	60.9	17.0
			All	2,141	75.1%	59.4	41.6	12.3
		
		Women	Unmarried	2,349	36.5%	9.3	9.3	2.7
			Married	3,777	94.9%	82.2	70.3	9.7
			All	6,128	72.5%	68.1	58.5	8.3
		
	1999	Men	Unmarried	1,406	48.1%	-.-	7.9	3.6
			Married	1,203	99.4%	-.-	57.9	23.3
			All	2,609	71.8%	-.-	40.4	16.4
		
		Women	Unmarried	2,354	38.9%	-.-	2.4	2.4
			Married	3,553	99.0%	-.-	43.7	13.8
			All	5,907	75.0%	-.-	35.1	11.4

The results from the 1994 Zimbabwe survey show that the three estimation methods yield very different estimates of the annual number of sex acts. Estimates based on the self-reported number of sex acts in the past four weeks (*F*_1_) give the highest estimates. Using this method, it is estimated that in 1994, sexually active unmarried males in Zimbabwe had 21 sex acts per year, while sexually active married men had 82 sex acts per year. For females, the number of sex acts is estimated at 9 per year for unmarried females and 82 for married females. This latter finding is fairly consistent with Brown (2002), who estimated the coital frequency for sexually active married women at 7.9 acts per month, which translates into 95 acts per year.

The second estimation method (*F*_2_), which is based on the proportion of respondents who reported having intercourse the day before the interview, results in an estimate of 8 sex acts per year for unmarried males, 61 for married males, 9 for unmarried females, and 59 for married females. Thus, this estimate consistently yields a lower estimate of the number of sex acts than the estimate based on the self-reported frequency of intercourse. This difference appears to be especially large for unmarried males.

The third estimation method (*F*_3_), which is based on a survival analysis using the assumption of a constant hazard, yields substantially lower estimates of the per capita annual number of sex acts. For unmarried males, the annual number of sex acts is estimated at only 4, while for married males it is estimated at 17. For females, the corresponding numbers are 3 and 10 per year, respectively. These estimates do not appear to be realistic.

For all other surveys examined here, we can also compare the estimates based on the proportion reporting intercourse the day before the survey (*F*_2_) and those based on the survival analysis with the assumption of a constant hazard (*F*_3_). The results confirm that this latter method consistently yields very low estimates of the number of sex acts. For example, among sexually active married males, the estimate of the annual number of sex acts ranges from 7.2 coital acts per year in the 1999 Nigeria survey to 23.3 in the 1999 Zimbabwe survey. For sexually active married females, the range is from 4.6 to 13.8, again in those same surveys. In other words, the results from the survival analysis using the assumption of a constant hazard suggest that in several countries, even married couples have intercourse less than once per month. Method F2 tends to yield higher estimates of the annual number of sex acts, but for both the 1998 Kenya and 1999 Nigeria surveys these estimates are also unrealistically low. In these latter cases, the low estimates are due to the fact that the number of respondents reporting last having intercourse the day before the survey is considerably lower than the number reporting last having intercourse two days ago.

The results based on the survival analyses appear unrealistic and are inconsistent with the published literature on the frequency of intercourse. For example, a study on coitus in sub-Saharan Africa estimates that the monthly coital frequency among sexually active married women ranges from 3.0 in Ghana to 8.1 for Rwanda [37], which corresponds with an annual frequency of 36 and 97 acts, respectively. Similarly, another study estimates the monthly coital frequency among married women at 6.1 act for Burundi, 3.0 for Kenya, and 5.7 for Uganda. Only Ghana has a substantially lower frequency of intercourse, at an average of 1.2 per coital acts per month [25]. The same study estimates that monthly coital frequency in Latin America ranges from 3.2 in Mexico to 8.0 in Brazil. A study on sexual activity among young women in Africa estimates the average number of sex acts in the past four weeks among women aged 15–24 in Kenya at 1.9 for the never married, and at 4.0 for the married. The corresponding data for Ghana are 0.7 and 1.0, respectively [29]. Hence, there is reason to believe that the results from the survival analysis are unreliable. (It is noteworthy that the results for Nigeria are substantially lower than those for the other countries, for both *F*_2 _and *F*_3_, largely because a substantially lower percentage of respondents reported having intercourse the day before they survey. Since the percentage reporting intercourse on other days is more in line with the results from the surveys in other countries, we suspect that this inconsistency is the result of a coding error.)

It is important to note that the results of the survival analyses are greatly affected by the type of decay function selected. Preliminary analysis using a Weibull decay function yielded estimates of the annual number of sex acts that are roughly one and a half to two times as high as estimates based on the exponential decay function proposed by Slaymaker and Zaba [28]. Unfortunately, determining which decay function to use requires information on the distribution of the length of the interval between two successive coital acts, and such information is not available in the DHS surveys.

### Probability of condom use

The estimates of the probability of condom use are shown in Table 3. As before, the three estimates of the probability of condom use could be calculated only for the 1994 Zimbabwe survey. Moreover, since the self-reported frequency of condom use was coded as "each time," "sometimes," or "never," we estimated the frequency on the basis of the proportion of each of these categories who reported using a condom in last intercourse. Thus, the estimates for *p*_1 _and *p*_2 _are nearly identical (although some differences exist when differentiating by marital status).

**Table 3:** Estimated probability of condom use per sex act

Country	Year	Sex	Marital Status	N of Cases	Estimation Method		
					
					Self-Reported Frequency of Use (*p*_1)_	Proportion Using at Last Intercourse (*p*_2)_	Proportion Using Day Before Interview (*p*_3)_
Kenya	1998	Men	Unmarried	1,644	-.-	40.8%	40.3%
			Married	1,763	-.-	9.1%	4.9%
			All	3,407	-.-	21.1%	18.3%
		
		Women	Unmarried	3,034	-.-	17.2%	0.0%
			Married	4,847	-.-	5.2%	3.0%
			All	7,881	-.-	7.7%	2.4%

Nigeria	1999	Men	Unmarried	1,072	-.-	39.2%	0.0%
			Married	1,608	-.-	6.1%	9.2%
			All	2,680	-.-	14.6%	6.9%
		
		Women	Unmarried	4,002	-.-	22.1%	7.9%
			Married	5,808	-.-	2.9%	5.4%
			All	9,810	-.-	5.8%	5.8%

Tanzania	1996	Men	Unmarried	985	-.-	34.5%	15.9%
			Married	1,268	-.-	5.5%	2.3%
			All	2,256	-.-	13.3%	6.0%
		
		Women	Unmarried	2,715	-.-	16.1%	6.2%
			Married	5,404	-.-	2.0%	1.0%
			All	8,120	-.-	4.2%	1.8%
		
	1999	Men	Unmarried	1,544	-.-	33.1%	23.4%
			Married	1,998	-.-	7.9%	3.2%
			All	3,542	-.-	15.7%	9.5%
		
		Women	Unmarried	1,421	-.-	20.6%	7.5%
			Married	2,608	-.-	3.8%	3.4%
			All	4,029	-.-	7.3%	4.3%

Zimbabwe	1994	Men	Unmarried	1,126	46.0%	53.6%	35.7%
			Married	1,015	13.9%	12.1%	6.8%
			All	2,141	25.8%	27.5%	17.5%
		
		Women	Unmarried	2,349	31.8%	30.7%	19.1%
			Married	3,777	5.6%	5.9%	5.0%
			All	6,128	10.7%	10.7%	7.7%
		
	1999	Men	Unmarried	1,406	-.-	65.6%	63.6%
			Married	1,203	-.-	8.5%	5.1%
			All	2,609	-.-	28.5%	25.5%
		
		Women	Unmarried	2,354	-.-	32.6%	19.7%
			Married	3,553	-.-	4.4%	1.9%
			All	5,907	-.-	10.3%	5.6%

When we compare the different methods to estimate the likelihood of condom use we notice that in the overwhelming number of cases the estimates based on the proportion reporting condom use at last intercourse of those who reported sex on the day before the interview (*p*_3_) are lower than those based on the data from the last sex act (*p*_2_). For example, in the 1999 Tanzania survey, the proportion who used a condom in last intercourse is 15.7% for males and 7.3% for females. By contrast, of those who had sex the day before the interview, the proportion who used a condom is only 9.5% and 4.3%, respectively. In part, these low estimates of *p*_3 _appear to stem from the fact that only a small number of survey respondents reported having intercourse the day before the interview. Consequently, there are some age groups where none of the respondents reported using a condom (not shown), which substantially lowers the estimate of the overall probability of condom use.

The results shown in Table 3 also indicate that the likelihood of having used condoms is substantially higher among unmarried than among married respondents. This finding is consistent with the literature [7,28,30,32] and thus confirms that our stratification by marital status was necessary, as the two groups also substantially differ in frequency of intercourse.

As other authors also have noted, women tend to report a much lower likelihood of condom use than men [21,31,32]. For example, Table 3 shows that in the 1999 Zimbabwe survey 29% of men but only 10% of women reported using a condom in last intercourse. Similarly, in the 1998 Kenya survey, 21% of men but only 8% of women reported using a condom in last intercourse. These differences persist when differentiating by marital status.

It is noteworthy that some gender discrepancies in the probability of condom use would be expected because African men may have sexual partners who are substantially younger. If the age difference between partners explained the gender differential in the probability of condom use, then we would expect that the probability of condom use for males aged 30–34 should be closer to that of women aged 25–29 or 20–24. Several data sets show that these probabilities are indeed closer, but the differences remain very large [21,31]. As most condoms are used in heterosexual sex acts, this discrepancy constitutes a serious problem when estimating overall condom use, because there is no way of verifying which of the two estimates provides the best estimate of the true probability of condom use.

### Estimated annual number of condoms used

Table 4 shows the estimates of the total annual number of condoms used based on different combinations of estimates for the frequency of intercourse and the probability of condom use. To facilitate interpretation, the bottom panel of the table also provides the highest and lowest estimates. For comparison, we also added data on the reported number of condom sales in the survey year, and in the year prior to the survey.

**Table 4:** Estimated annual number of condoms used

Estimation Method		Kenya 1998	Nigeria 1999	Tanzania 1996	Tanzania 1999	Zimbabwe 1994	Zimbabwe 1999
						
Frequency of Intercourse	Probability of Condom Use						
							
**Males**							
							
F_1 _Self-Reported	p_1 _Self-Reported	-.-	-.-	-.-	-.-	18,047,620	-.-
	p_2 _Last Intercourse	-.-	-.-	-.-	-.-	19,451,694	-.-
	p_3 _Previous Day	-.-	-.-	-.-	-.-	11,408,033	-.-
							
F_2 _Previous Day	p_1 _Self-Reported	-.-	-.-	-.-	-.-	12,209,655	-.-
	p_2 _Last Intercourse	10,650,977	5,522,394	14,919,839	19,053,896	11,515,528	10,850,758
	p_3 _Previous Day	7,734,312	6,779,088	6,231,789	9,805,457	6,275,443	7,660,061
							
F_3 _Survival Analysis	p_1 _Self-Reported	-.-	-.-	-.-	-.-	4,136,103	-.-
	p_2 _Last Intercourse	10,121,645	18,858,423	4,891,365	7,493,313	3,999,271	4,468,660
	p_3 _Previous Day	7,221,404	10,010,100	2,439,635	3,754,680	2,324,967	3,262,927
							

							
**Females**							
							
F_1 _Self-Reported	p_1 _Self-Reported	-.-	-.-	-.-	-.-	7,980,256	-.-
	p_2 _Last Intercourse	-.-	-.-	-.-	-.-	8,406,142	-.-
	p_3 _Previous Day	-.-	-.-	-.-	-.-	7,088,876	-.-
							
F_2 _Previous Day	p_1 _Self-Reported	-.-	-.-	-.-	-.-	6,913,439	-.-
	p_2 _Last Intercourse	3,375,708	4,632,093	5,529,321	10,744,128	7,253,275	3,700,789
	p_3 _Previous Day	2,091,845	7,622,258	2,759,809	8,422,675	6,115,040	1,591,401
							
F_3 _Survival Analysis	p_1 _Self-Reported	-.-	-.-	-.-	-.-	1,111,439	-.-
	p_2 _Last Intercourse	2,200,502	4,503,194	993,705	2,756,648	1,137,474	1,395,517
	p_3 _Previous Day	986,769	5,253,132	444,480	1,994,578	914,083	647,804
							

							
Highest Estimate		10,650,977	18,858,423	14,919,839	19,053,896	19,451,694	10,850,758
Lowest Estimate		986,769	4,503,194	444,480	1,994,578	914,083	647,804
							
Sales, Survey Year		11,797,536	108,444,464	41,629,132	45,024,836	38,316,656	71,432,882
Sales, Previous Year		13,516,931	67,629,732	51,030,840	53,409,352	63,778,992	35,751,329
							

The results presented in Table 4 indicate that the methodologies yield radically different estimates of the total number of condoms used. This was anticipated, considering that our estimates of the frequency of intercourse and the probability of condom use also varied by estimation method. There are also very large differences between the estimates based on data from the female surveys and those from the male surveys. The bottom panel of Table 4 shows that the range of the estimates is very wide for all surveys. For example, in Kenya the high estimate of the total annual number of condoms used in 1998 is 10.7 million, while the low estimate is only 1.0 million. Similarly, for the 1999 Tanzania survey the highest estimate is 19.1 million while the lowest estimate is only 2.0 million.

It is unknown which of the estimates is most accurate. However, as we previously noted, the *p*_3 _estimate (which is based on condom use among those who reported having intercourse the day before the survey) appears unreliable due to the small number of cases. In addition, the survival analyses yielded unrealistically low estimates of the frequency of intercourse (*F*_3_) that appeared inconsistent with the literature. Therefore, estimates that are based on these two factors are unlikely to be reliable. Table 4 confirms that estimates based on *F*_3 _and *p*_3 _usually yield the lowest estimates of the total number of condoms used.

When self-reported data are not available, estimates based on *F*_2 _and *p*_2 _are likely to be the most reliable. Data from the 1994 Zimbabwe survey confirm that the estimates based on the self-reported frequency of intercourse (*p*_1_) and the percentage who used a condom in last intercourse (*p*_2_) yield fairly similar results. This was anticipated, given that self-reported frequency of intercourse was coded as a categorical variable and subsequently quantified on the basis of the percentage who reported using a condom in last intercourse. Table 4 shows that estimates based on *F*_1 _and *F*_2 _are also fairly close.

Nevertheless, all survey-based estimates of the annual number of condoms used are substantially lower than the reported number of condoms sold for almost every country. The only exception is Kenya, where the high estimate of the total number of condoms used based on the 1998 Kenya DHS is fairly close to the number distributed (10.7 million vs. 11.8 million). For the other surveys, the reported number of condoms sold or distributed tends to be 2.5 to 3.0 times higher than even the highest survey-based estimate of the number of condoms used. Comparison with sales data from the previous year does not resolve these differences.

## Conclusions

The purpose of this paper was to estimate the annual number of sex acts and condoms used based on survey data, and to compare the latter with data on the annual number of condoms sold and distributed. The ability to estimate the number of sex acts from survey data would be a valuable tool for program managers, as it would enable them to estimate the number of condoms needed. Since the available data on condom sales and distribution measure the number of condoms supplied to the trade rather than to the consumer, survey estimates of the total number of condoms used could also help clarify to what extent data on the number of condoms supplied to the trade reflects actual consumer sales.

Analysis of the annual reported number of condoms sold and distributed reveals very erratic patterns. The large year-to-year differences in the total number of condoms distributed clearly do not reflect differences in the number of condoms sold to consumers, nor in the level of condom use, as this would imply major changes in behavior. The latter is unlikely to have occurred, since behavior is known to change very slowly. In other words, the large fluctuations in the number of condoms provided to the trade are likely to reflect fluctuations in condom inventory at various levels in the distribution chain. Because of this, the current data on the number of condoms sold and distributed say very little, if anything, about the number of condoms sold to consumers or about actual levels of condom use.

To estimate the annual number of condoms used from survey data, survey questionnaires would ideally ask respondents how often they had sex during a given reference period and how often they used a condom during that period. Considering that using very long reference periods (e.g., a year) is likely to cause recall errors, a shorter reference period is preferable. Of the DHS studies used in this paper, only one (Zimbabwe DHS-III, 1994) asked respondents about the frequency of intercourse during the four weeks preceding the survey. For the other surveys, the frequency of intercourse had to be estimated indirectly on the basis of the duration since last intercourse. Although older data on frequency of intercourse are available for some countries, such data may not provide reliable estimates of current behavior, as the HIV/AIDS crisis and other factors may have influenced sexual behavior.

If future surveys are to estimate the annual number of condoms used, then questions enquiring about the total number of sex acts and the total number of sex acts in a fixed time period should be added. For example, recent surveys in Zambia asked about the number of sex acts and the number of condoms used in the past week, which can easily be extrapolated to a one-year period [33]. Asking about the timing of the last two sex acts, rather than only the very last sex act, would also be recommended. This would provide data on the duration between two successive sex acts, which will improve estimation of the total number of sex acts using survival methodologies. Knowing the distribution of the time interval between successive sex acts would also enable researchers to identify a decay function that best fits the data, which will substantially increase the accuracy of the estimates.

The results of our survey analyses, which are based on DHS data currently available, show that the estimates of both the number of sexual acts and the number of condoms used vary enormously based on the estimation method used. For several surveys, the highest estimate of the annual number of condoms used is tenfold that of the lowest estimate. While some estimation methods can be disregarded because they yield results that are clearly not plausible, it is impossible to determine which of the remaining methods yield the most accurate results. Until the reliability of these various estimation methods can be established, estimating the annual number of condoms used from survey data will not be feasible.

To be able to verify the reliability of the estimates of the number of condoms used, it is necessary to have accurate data on the number of condoms sold and distributed to consumers. In developing countries, such is not feasible, in part due to the lack of standardized record-keeping, and because many condoms are distributed through informal retailers, such as street venders and hawkers, who are unlikely to keep records. For the purpose of testing the feasibility of the estimation methods, it may therefore be more productive to use data from developed countries where retail-level condom sales data are available (assuming such data are not proprietary). Alternatively, it may be possible to test the reliability of the estimates in developing countries, by obtaining the relevant sales data on a smaller scale (e.g., for one district only). However, sales data have the drawback that they do not provide information about the characteristics of the consumers. Consequently, sales data are unable to provide detailed information about program impact.

## Competing interests

The author(s) declare that they have no competing interests.

## Authors' contributions

DM conceived of the study and drafted the manuscript. RVR developed the study design and carried out the statistical analysis. Both authors read and approved the final manuscript.

## Pre-publication history

The pre-publication history for this paper can be accessed here:



## Supplementary Material

Additional File 1 This file contains the background data for the calculations Click here for file
