# Supplementary material for: Explaining inconsistencies between data on condom use and condom sales
Source: BMC Health Serv Res. 2005 Jan 15;5:5. doi: 10.1186/1472-6963-5-5 (PMC545997; doi:10.1186/1472-6963-5-5)
Supplement: Additional File 1 — This file contains the background data for the calculations [file 1472-6963-5-5-S1.doc]

Appendix: Background data for calculations

| Country | Year | Sex |  | | | | |
| --- | --- | --- | --- | --- | --- | --- | --- |
|  |  |  | Population Size^a^ | % Covered by Survey^b^ | % Sexually  Active^c^ |  |  |
| Kenya | 1998 | Men | 14,048,888 | 44.9% | 82.7% |  |  |
|  |  | Women | 14,667,582 | 43.8% | 73.0% |  |  |
|  |  |  |  |  |  |  |  |
| Nigeria | 1999 | Men | 62,343,422 | 48.7% | 72.4% |  |  |
|  |  | Women | 61,523,461 | 46.6% | 67.8% |  |  |
|  |  |  |  |  |  |  |  |
| Tanzania | 1996 | Men | 14,705,131 | 43.6% | 70.6% |  |  |
|  |  | Women | 15,774,725 | 43.1% | 67.8% |  |  |
|  |  |  |  |  |  |  |  |
|  | 1999 | Men | 15,641,498 | 43.8% | 80.5% |  |  |
|  |  | Women | 17,279,312 | 44.3% | 79.2% |  |  |
|  |  |  |  |  |  |  |  |
| Zimbabwe | 1994 | Men | 5,513,912 | 44.5% | 75.1% |  |  |
|  |  | Women | 5,740,749 | 44.7% | 72.5% |  |  |
|  |  |  |  |  |  |  |  |
|  | 1999 | Men | 6,102,041 | 47.9% | 71.8% |  |  |
|  |  | Women | 6,286,279 | 45.7% | 75.0% |  |  |
|  |  |  |  |  |  |  |  |

1. Estimate based on sex distribution in the DHS
2. Estimate based on age distribution in the DHS
3. Estimate based on DHS data
